# A prospective phase I multicentre randomized cross-over pharmacokinetic study to determine the effect of food on abiraterone pharmacokinetics

**DOI:** 10.1007/s00280-019-03952-w

**Published:** 2019-09-12

**Authors:** Floor J. E. Lubberman, Guillemette E. Benoist, Winald Gerritsen, David M. Burger, Niven Mehra, Paul Hamberg, Inge van Oort, Nielka P. van Erp

**Affiliations:** 1 Department of Pharmacy (864), Radboud Institute for Health Sciences, Radboud University Medical Center, PO Box 9101, 6500 HB Nijmegen, The Netherlands; 2 Department of Medical Oncology, Radboud Institute for Health Sciences, Radboud University Medical Center, Geert Grooteplein Zuid 10, 6525 GA Nijmegen, The Netherlands; 3 Department of Medical Oncology, Franciscus Gasthuis and Vlietland, Kleiweg 500, 3045 PM Rotterdam, The Netherlands; 4 Department of Urology, Radboud Institute for Health Sciences, Radboud University Medical Center, Geert Grooteplein Zuid 10, 6525 GA Nijmegen, The Netherlands

**Keywords:** Abiraterone, Bioequivalence, Pharmacokinetics, Food, Prostate cancer

## Abstract

**Purpose:**

Abiraterone acetate is used at a fixed oral dose of 1000 mg once daily (OD) taken fasted. By administering abiraterone acetate with food, a reduced dose can potentially be given while maintaining equivalent abiraterone exposure. Moreover, administering abiraterone acetate with a breakfast is considered more patient friendly. The aim of this study was to establish the bio-equivalent lower dose of abiraterone when taken with a continental breakfast (CB) compared to the standard intake of 1000 mg OD fasted.

**Methods:**

In this phase I, randomized cross-over, multi-center study, abiraterone pharmacokinetics (PK) were evaluated in patients with metastatic castration-resistant prostate cancer who were treated for 14 days with 1000 mg abiraterone acetate taken fasted, followed by 14 days of treatment with 500 mg taken with a CB.

**Results:**

14 patients were enrolled into the study, of whom 12 were eligible for PK analysis. The geometric mean ratio (GMR) (fed/fasted) was 0.88 (90% CI 0.73–1.07) for area-under-the-curve (AUC_0–24h_), 1.03 (90% CI 0.79–1.34) for *C*_max_ and 0.81 (90% CI 0.60–1.10) for *C*_trough_, respectively. High inter-patient variability (> 50%) was found for all PK parameters under both intake conditions. Patients seemed to be slightly more satisfied about the intake of 500 mg abiraterone acetate when taken with a CB compared to 1000 mg fasted.

**Conclusion:**

In conclusion, a bioequivalent lower dose of abiraterone taken with food could not be established in our study. Although based on the absence of a exposure–toxicity relationship, the strict bioequivalence margins as defined by the FDA guidelines could be applied more flexible for abiraterone. Information on the effect of food on abiraterone pharmacokinetics as presented in our study can be used for patients with difficulties taken their medication fasted.

**Electronic supplementary material:**

The online version of this article (10.1007/s00280-019-03952-w) contains supplementary material, which is available to authorized users.

## Introduction

With the introduction of oral targeted anti-cancer drugs, the intake of food as a cause of intra-patient variability in drug absorption has become relevant. As part of the registration process of these new drugs, studies to quantify the effect of food on pharmacokinetics are routinely performed. When food affects drug absorption, the advised intake regime is often fasted for oncologists [1].

One of the drugs with an outspoken food effect is abiraterone acetate (AA) (Zytiga^®^). AA is a pro-drug of abiraterone. Abiraterone is a selective inhibitor of cytochrome (CYP) 17A1, a crucial enzyme in androgen biosynthesis, resulting in virtually undetectable serum and intra-tumor androgen levels and thereby resulting in antitumor activity in patients with locally advanced or metastatic prostate cancer (mHSPC and mCRPC) [2–4]. AA tablets are administered at a fixed oral dose of 1000 mg OD in a fasted state in combination with 10 mg prednis(ol)one daily [5].

Abiraterone acetate was developed to overcome the poor bioavailability of the initially formulated abiraterone. Nevertheless, also AA shows very limited bioavailability mainly due to its physiochemical properties of low solubility in aqueous media and low permeability properties [6]. The bioavailability of AA is majorly affected by ingestion with food. However, large differences in the influence of food on pharmacokinetics of AA are seen between different studies. Chi et al. showed that the intake of AA 1000 mg with a low-fat FDA meal in healthy volunteers resulted in a sevenfold increase in *C*_max_ and a fivefold increase in AUC_0–∞_ compared to fasted intake. Whereas, with a high-fat FDA meal a 17-fold increase in *C*_max_ and a tenfold increase in AUC_0–∞_ was seen [7]. Surprisingly, a more modest difference was seen when mCRPC patients ingested 1000 mg AA with a low-fat FDA meal compared to modified fasted intake. The geometric mean ratios (GMRs) fed versus fasted were only 1.35 for *C*_max_ and 1.07 for AUC_0–24h_ [7]. In another study by Attard et al., AA capsules administered with high-fat food in mCRPC patients resulted in a 4.4-fold increased AUC_0–∞_ [8]. Despite these large differences, the overall picture suggests that AA is much better absorbed in the presence of food. This effect is most likely due to the better solubility of AA in the presence of food, of which the amount of fat in the meal is thought to be the most important [7]. The clinical potential of food to reduce the required dose of AA was shown by Szmulewitz et al. They showed that a reduced dose of 250 mg AA taken with a low-fat breakfast was non-inferior compared to the standard dose of 1000 mg taken fasted for the surrogate endpoint (> 50% PSA response) [9].

Since the effect of food on abiraterone PK is not fully established, a bioequivalence study comparing fasted versus fed intake could contribute to the understanding the effect of food on abiraterone pharmacokinetics. In addition, ingestion of drugs with a breakfast is easier to implement in the patient’s daily life and preferred by most [10]. Consequently, patients are less likely to forget their medication and therefore, an intake regimen with food might positively affect drug adherence.

Therefore, in this study, we aimed to determine the effect of a continental breakfast (CB) on abiraterone exposure and establish the dose reduction required to reach a bio-equivalent exposure compared to the registered intake of 1000 mg OD without food.

## Methods

### Study design and participants

For this multicentre, phase 1, cross-over study, we enrolled patients from two investigational sites in the Netherlands (Franciscus Gasthuis and Vlietland and Radboud university medical center). Eligible patients were aged 18 or older, had metastatic castration-resistant prostate cancer, were treated with 1000 mg abiraterone acetate (Zytiga^®^) OD fasted (both patients on treatment and patients who started treatment were eligible) and had an Eastern Cooperative Oncology Group (ECOG) performance status of 0–2 [5]. Patients with gastrointestinal abnormalities that could influence the absorption of AA were excluded. The use of other substances known or likely to interfere with the pharmacokinetics of abiraterone was prohibited during this study.

The trial was approved by the Investigational Review Board of Radboud university medical center, Nijmegen, the Netherlands. The trial was conducted in accordance with Good Clinical Practice and the Declaration of Helsinki and registered at ClinicalTrials.gov, number NCT02883166. All patients gave written informed consent before entering the study.

### Procedures

**Fig. 1:**
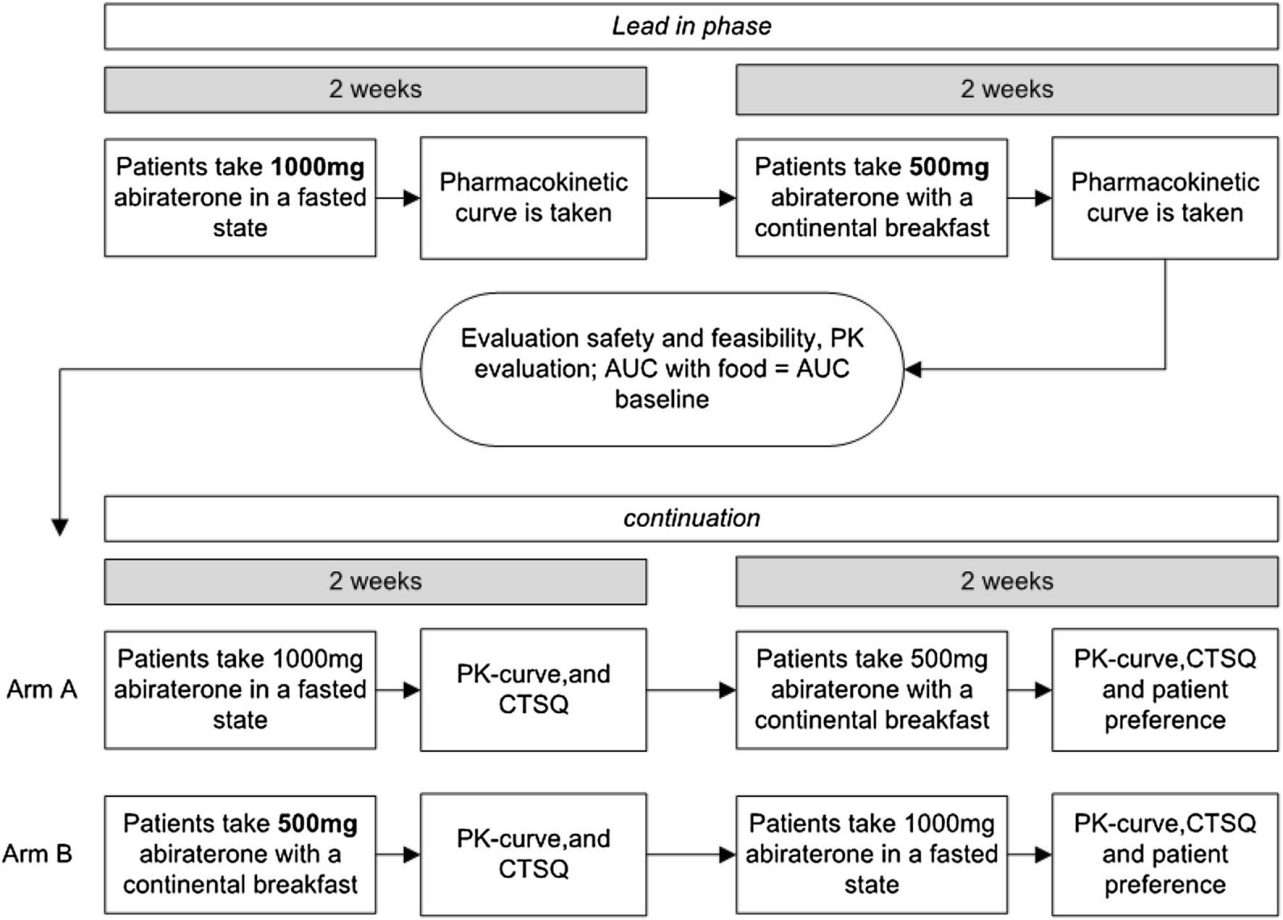
Study design

Since the effect of a CB on abiraterone exposure was uncertain and the study was conducted in patients with cancer, a lead-in phase of three patients was introduced. Based on the data of Chi et al., at least a twofold increase in abiraterone exposure taken with food was presumed [7]. Therefore, patients received 500 mg AA with CB. Pharmacokinetic (PK) evaluation after 2 weeks was conducted to prevent unnecessary over- or under-dosing. During the first 2 weeks, patients took 1000 mg AA OD in a fasted state at 08:00 am followed by one of the standardized CBs (Table 1) at 09:00 am. After reaching steady-state pharmacokinetics (day 14), blood was collected in K2-EDTA tubes at *t* = 0, 1, 2, 3, 4, 5, 6, 8, 10, 12 and 24 h after AA intake for PK assessment. Subsequently, these patients switched to 500 mg AA OD (50% dose reduction) in combination with a standardized CB at 08:00 am. At day 28 of the study, after reaching steady-state pharmacokinetics, the second PK assessment was performed. The results of the first 3 patients were analyzed and evaluated before continuation. If the GMR of the AUC_0–24h_ and *C*_max_ of the reduced dose taken with food compared to the 1000 mg fasted appeared to be within the threshold for bioequivalence (0.8–1.25), the next 21 patients would be exposed to the 50% dose reduction with food. When the 50% dose reducing strategy in the lead-in phase led to a ratio of AUC_0–24h_ and *C*_max_ of more than 1.25, a 75% dose reduction (i.e., 250 mg) would be tested for bioequivalence in a next lead-in of 3 new patients (Fig. 1).

**Table 1:** Breakfast composition

Option 1
1 slice of wheat bread
Greased with diet margarine
Topped with luncheon meat or cheese
1 glass of tea
Option 2
350 mL full cream vanilla custard
Option 3
2 crackers golden brown
Greased with diet margarine
1 topped with low-fat meat
1 greased with jam
1 glass of tea
Option 4
1 slice of wheat bread
Greased with diet margarine
Topped with milk chocolate sprinkles
1 Beaker semi-skimmed milk
Option 5
300 mL full- fat milk
3 Spoons of sugar
30 g bambix 8-wheat breakfast 15 months
Option 6
150 mL full-fat yogurt
40 g muesli with sugar
1 glass of tea
Option 7
1 slide of wheat bread
Greased with peanut butter
1 glass of tea

All breakfasts contain the same amount of fat namely 9–10 g

After each treatment period, patients were asked to complete the validated cancer therapy satisfaction questionnaire (CTSQ) [11]. In the CTSQ, patients are asked about their feelings about side effects, satisfaction with therapy and their expectations of therapy. A difference of ≥ 8.3 points in patients expectations of therapy (ET), ≥ 5.9 point in patients satisfaction with therapy (SWT) and ≥ 10.3 for patients feelings about side effect (FSE) were considered clinically relevant [12].

The CBs were composed by a dietician from Radboudumc and designed to be similar to the breakfasts our patients normally would have. All proposed breakfasts contained the same amount of fat (9–10 g, 25–50% of total caloric intake). The total amount of calories, proteins and carbohydrates differed per breakfast, ranging , respectively, 160–320 Cal, 5–11 g and 15–50 g (Table 1). The choice of CB was at patients’ discretion.

Blood samples for measurement of abiraterone PK were collected on ice and centrifuged within 1 h at 1900*g* for at least 5 min (4 °C). Plasma samples were stored at − 40 °C until the day of analysis. Abiraterone plasma concentrations were measured using a validated liquid chromatography tandem mass spectrometry method with a range of 1–500 mg/L [13].

### Outcomes

The primary endpoint of this study was to determine the equivalent reduced dose of AA when taken with a CB compared to the recommended intake of AA 1000 mg OD taken fasted. Bioequivalence is assumed when the geometric mean ratio (GMR) including the 90% CI of the AUC_0–24h_, *C*_max_ and *C*_trough_ is within the thresholds of 0.80 and 1.25. Due to the mild toxicity profile of abiraterone (e.g., no treatment-related grade 3 or 4 toxicities occurred at doses twice the registered dose), CI crossing the upper limit of 1.25 is accepted [8].

### Statistical analysis

Based on an intra-patient coefficient of variation (CV) of 25%, and a reference ratio of 1.0, a sample size of 24 patients was required for a power of 80%, a two-side significance level of 0.05 and a CV of 20% on the log-transformed data [14]. The AUC_0–24h_, *C*_max_, *C*_trough_, were calculated using non-compartmental analyses in WinNonlin/Phoenix version 6.3 (Pharsight Corporation^®^). The CTSQ questionnaires were scored following the guideline provided by Abetz et al. [12]. The differences in CTSQ scores were analyzed according to Altman et al. to correct for a possible period effect [15].

## Results

Between November 2016 and January 2019, a total of 14 Caucasian patients treated with AA for metastatic castration-resistant prostate cancer were enrolled in the study, of which 12 were evaluable for pharmacokinetic analysis. One patient stopped AA therapy before the second PK evaluation due to elevation in liver enzymes and one patient did not take the medication as prescribed. Both patients were, therefore, excluded from analysis. Of the 12 eligible patients, 10 received prior systemic treatment. Two patients were treatment naïve. Eight patients already used AA before study participation. The median age of the patients was 70 (range 64–93) years (Table 2).

**Table 2:** Patient characteristics at baseline

Characteristics	No.	%
Patients	12	100
Age, year		
Median (range)	70 (64–93)	
Ethnics		
Caucasian	16	100
BMI, kg/m^2^		
Median (range)	29 (21–37)	
ECOG performance status		
0	6	50
1	6	50
Received previous treatment		
Yes	10	83
No	2	17
PSA (ng/mL)		
Median (range)	17 (0.2–93)	
Hemoglobin (mmol/L)		
Median (range)	8.2 (6.4–9.4)	
LDH (ng/mL)		
Median (range)	211 (179–280)	

*ECOG* Eastern Cooperative Oncology Group

In the initial lead-in phase of the study, three patients were treated with a reduced dose of 500 mg OD with a CB. These three patients showed a geometric mean (GM) AUC_0–24h_ value with and without food of 595 mg h/L and 598 mg h/L, respectively. The GM *C*_max_ values with and without food were 116 mg/L and 115 mg/L, respectively. The GMRs fed/fasted calculated in these patients were 1.01 and 0.99 for AUC_0–24h_ and *C*_max_. Based on these results, a reduced dose of 50% with a CB was studied in the following patients.

**Fig. 2:**
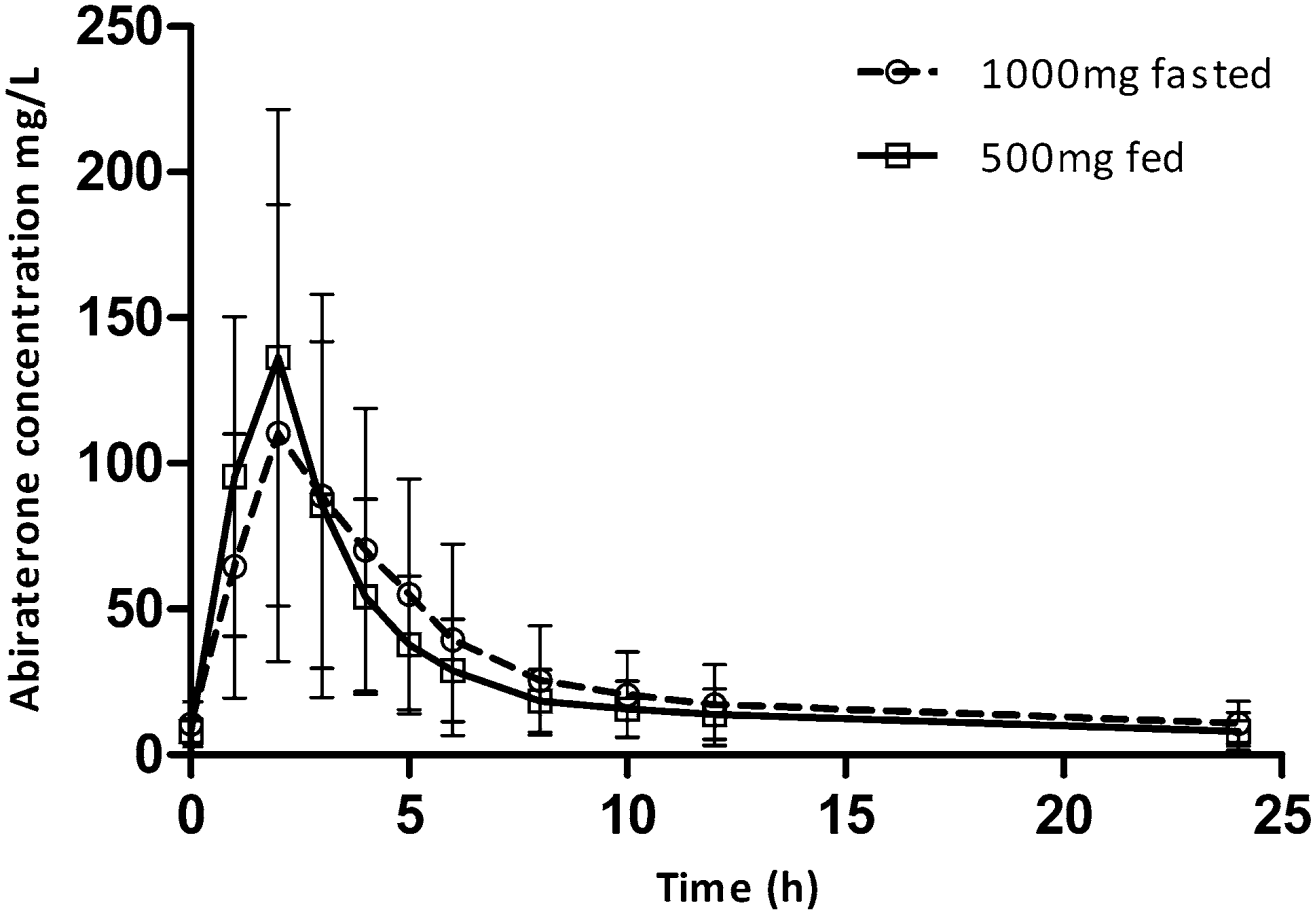
Concentration time curve of 1000 mg abiraterone acetate taken fasted and 500 mg abiraterone acetate taken with a continental breakfast

In the final PK analysis in 12 patients, the GM of the AUC_0–24h_ was 776 mg h/L and the *C*_max_ was 148 mg/L, respectively, when 1000 mg abiraterone was taken in a fasted state. When a reduced dose of 500 mg abiraterone was taken with a CB, the GM of the AUC_0–24h_ and the *C*_max_ was 686 mg h/L and 152 mg/L, respectively. The GM of the *C*_trough_ was 10.7 mg/L, when 1000 mg was taken fasted, compared to 8.7 mg/L, when 500 mg was taken with a CB (Fig. 2, Table 3).

**Table 3:** Pharmacokinetic parameters abiraterone

	1000 mg fasted	500 mg fed
AUC_0–24h_, mg h/L, GM (90 CI, CV%)	776 (514–1172)	686 (455–1035)
*C*_max_, mg/L, GM (90 CI, CV%)	148 (98–223)	152 (99–235)
*C*_trough_, mg/L, GM (90 CI, CV%)	10.7 (6.6–14.9)	8.7 (4.8–12.4)

*AUC*_*0–24h*_ area under the concentration time curve, *C*_*max*_ maximum observed plasma concentration, *C*_*trough*_ plasma concentration at *t* = 24 h, *GM* geometric mean, *CI* confidence interval, *CV%* percentage of coefficient of variation defined by (standard deviation/mean) × 100

**Fig. 3:**
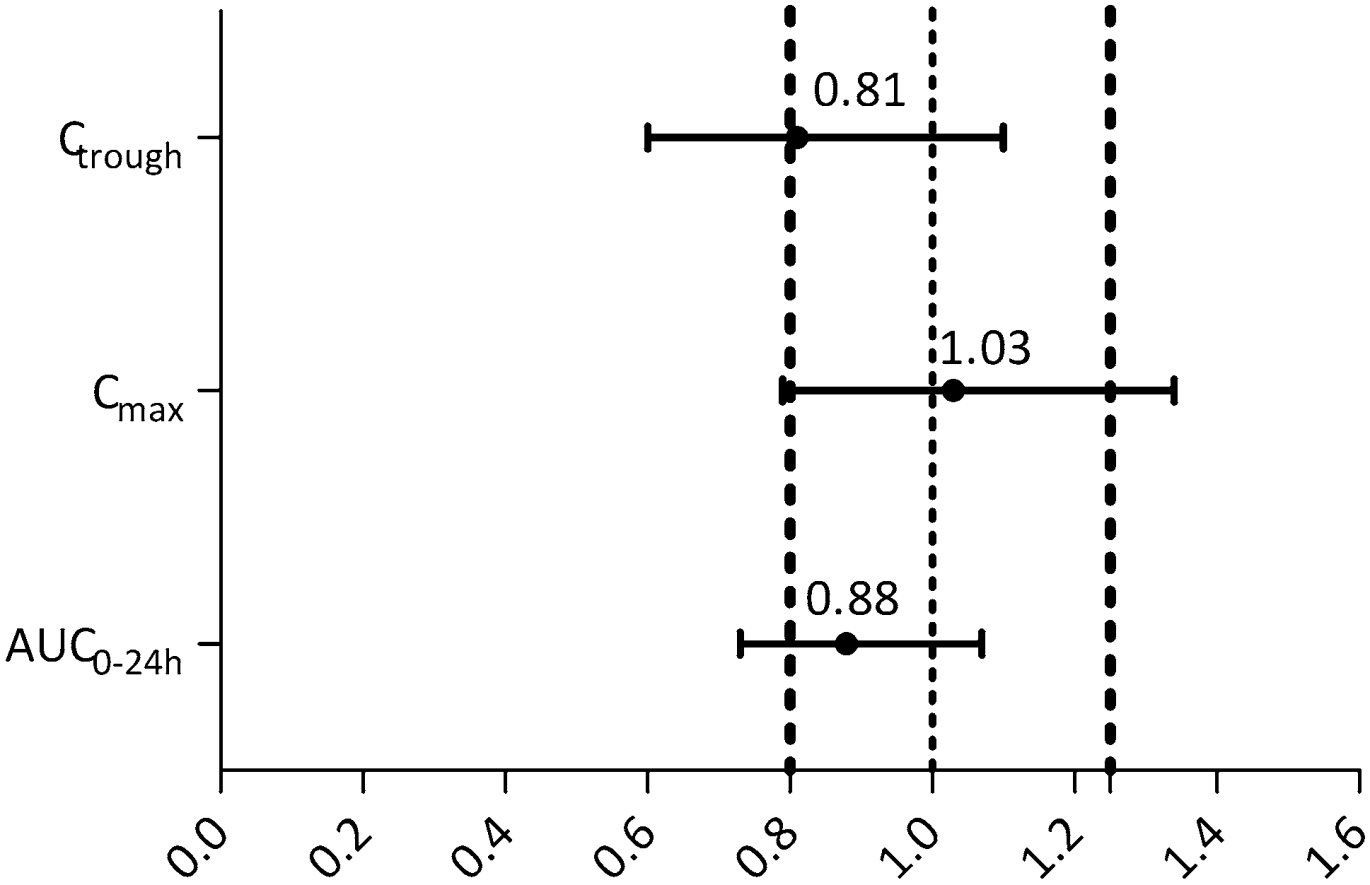
Geometric mean ratio including their confidence interval. Vertical lines represent the threshold of 0.8 and 1.25 as defined by the FDA

To determine the difference between both intake regimens on AUC_0–24h_, *C*_max_ and *C*_trough_ the GMRs fed/fasted were calculated including the 90% CI. For AUC_0–24h_ the GMR was 0.88 (90% CI 0.73–1.07), for *C*_max_ the GMR was 1.03 (90% CI 0.79–1.34) and for *C*_trough_ the GMR was 0.81 (90% CI 0.60–1.10). These GMRs with their 90% confidence intervals do not fall within the thresholds predefined for bioequivalence (0.80–1.25) (Fig. 3).

Due to the large variation in the individual GMRs observed in our study, we decided after 12 patients that bioequivalence could not be confirmed even when the predefined number of 24 patients would be met. Therefore, further enrolment was stopped.

Inter-patient variability (CV %) for AUC_0–24h_, *C*_max_, and *C*_trough_ was 65% vs. 57%, 55% vs. 57% and 72% vs. 75%, respectively, when abiraterone was taken fasted compared to fed.

Compared to 1000 mg taken fasted, patients seem to be slightly more positive about the intake of 500 mg abiraterone with a continental breakfast based on the differences in FSE, SWT and ET fasted versus fed of 2.8, 6.6 and 8.5 points, respectively (Table 4).

**Table 4:** CTSQ

	1000 mg fasted (*N* = …)	500 mg fed (*N* = …)
Expectations of therapy ET, mean (95% CI) (*n* = 11)	38.6 (28.2–49.1)	47.1 (35.6–58.5)
Feelings about side effects FSE, mean (95% CI) (*n* = 10)	76.0 (65.1–87.0)	78.8 (64.0–93.5)
Satisfaction with therapy SWT, mean (95% CI) (*n* = 10)	81.3 (72.5–90.0)	87.9 (80.5–95.3)

*CTSQ* cancer therapy satisfaction questionnaire, *CI* confidence interval

## Discussion

We aimed to establish the dose reduction required to reach a bioequivalent exposure compared to the standard intake of 1000 mg abiraterone acetate OD without food. Although the GMRs of the intake of 500 mg abiraterone acetate with a continental breakfast compared to 1000 mg taken fasted are within the thresholds of 0.80 and 1.25, the 90% confidence intervals do not meet the criteria for bioequivalence. However, it is known that no exposure–toxicity relation has been found for abiraterone when investigated up until doses of 2000 mg [8]. Therefore, the strict bio-equivalence margins as defined by the FDA guidelines could be applied more flexible for abiraterone.

To the best of our knowledge, this is the first study to investigate the bioequivalence of an adjusted AA dose when taken with food. Our data demonstrated that a 50% dose reduction resulted in a GMR for the AUC_0–24h_ and *C*_trough_ below 1.0. Based on our PK data, no further dose reduction was deemed feasible. However, Szmulewitz et al. have shown that a 75% reduced AA dose taken with a low-fat breakfast resulted in similar PSA response rates compared to the standard fasted intake of AA 1000 mg OD [9]. The descriptive PK data in their study demonstrated that the abiraterone *C*_trough_ levels were lower in the group treated at the reduced dose with food compared to the full dose taken fasted [9]. Nevertheless, despite the lower *C*_trough_ levels, the percentage of patients reaching an adequate PSA response remained comparable between both intake regimes [9]. The majority of the patients in the study of Szmulewitz et al. was treated prior to receiving chemotherapy and might, therefore, be more sensitive to lower abiraterone concentrations. Xu et al. showed that the EC50 of the PSA for abiraterone was 1.56 ng/mL in chemotherapy-naïve patients and 4.75 ng/mL in patients who underwent previous chemotherapy [16]. Though, due to the large confidence intervals around the observation of Xu et al., the data of those trials should be interpreted carefully. Our population mainly consisted of patients post chemotherapy, Therefore, exposure levels similar to those reached when 1000 mg AA taken fasted were aimed for. The results of our study are based on 12 patients instead of the predefined 24 patients. Variation of the individual GMRs of the AUC_0–24h_ and *C*_max_ was larger than expected. Re-estimation of the sample, size based on the larger variability as observed in our study, learned us that bioequivalence could not be demonstrated in the predefined number of 24 patients. Therefore we considered it unethical to further conduct the study. Nevertheless, as 12 patients are the minimal number necessary for a bioequivalence study as stated by the FDA, the PK results from our study are still of value for further interpretation [17]. Since we could not demonstrate bioequivalence, the switch of large groups of patients to an alternative intake regime of 500 mg taken with food cannot be supported. However, because the GMRs of the AUC_0–24h_, *C*_max_ and *C*_trough_ were within the threshold of 0.8–1.25, the data are suggestive for bioequivalence [18]. Therefore, in individual patients, intake of 500 mg with a CB accompanied with PK monitoring could be considered when experiencing difficulties with a fasted intake.

During our study, patients could choose between seven different types of breakfast. Though standardized for the amount of fat, differences in total caloric intake between the breakfasts were present. This could have enhanced the inter-patient variability. However, our results demonstrate that the inter-patient variability in our study was comparable to other data and was not increased when AA was ingested with food [16, 19]. It, therefore. is not expected that the differences in breakfast contribute to the inter-patient variability.

Our study is the first to explore patient satisfaction when abiraterone is taken with food as a clinical outcome. Our study indicates a modest improvement in treatment satisfaction and expectation of therapy when 500 mg of abiraterone was taken with food. The absence of any notable differences regarding patients feelings about side effects is likely due to the limited gastro-intestinal adverse events such as diarrhea or nausea that patients experience when treated with abiraterone (1–3% grade 3 or 4) [3]. Therefore, ingesting AA with food will not contribute to a reduction of these adverse events.

For several other drugs with this large intra- and inter-patient PK variability, the formulation was adjusted to increase drug absorption and drug exposure to achieve a better predictable response in patients. Examples of drugs with alternative formulations due to absorption issues are regorafenib and olaparib [20, 21]. Also for AA, a new formulation is tested, using a continuous flow precipitation technology. This new formulation shows improved bioavailability, and therefore less PK variability. In addition, food does not play a significant role in the absorption of this new abiraterone formulation [22]. We believe that this new formulation, which is not available yet, could help to overcome the issues of highly variable PK and thereby unpredictable treatment effect for patients.

In conclusion, a bioequivalent lower dose of abiraterone taken with food could not be established in our study. Though, information on the effect of food on abiraterone pharmacokinetics as presented in our study can be used.

## Electronic supplementary material

Below is the link to the electronic supplementary material.

Supplementary material 1 (DOCX 16 kb)
