# Supplementary material for: A prospective phase I multicentre randomized cross-over pharmacokinetic study to determine the effect of food on abiraterone pharmacokinetics
Source: Cancer Chemother Pharmacol. 2019 Sep 12;84(6):1179–85. doi: 10.1007/s00280-019-03952-w (PMC6820614; doi:10.1007/s00280-019-03952-w)
Supplement: Supplementary file 1 — Supplementary material 1 (DOCX 16 kb) [file 280_2019_3952_MOESM1_ESM.docx]

Supplementary 1

|  | | **Protein (g)** | **Protein (Kcal)** | **Fat**  **(g)** | **Fat**  **(Kcal)** | **Carbohydrates (g)** | **Carbohydrates (Kcal)** | **Total**  **Energy**  **(kcal)** |
| --- | --- | --- | --- | --- | --- | --- | --- | --- |
| **Option 1** | |  |  |  |  |  |  |  |
| 1 | Slice Wheat bread | 3 | 12 | 1 | 9 | 16 | 64 | 85 |
| 1 | with diet margarine | 0 | 0 | 4 | 36 | 0 | 0 | 36 |
| 1 | Topped with luncheon meat or cheese | 2 | 8 | 4,50 | 40,5 | 0 | 0 | 48,5 |
| 1 | Glass of tea | 0 | 0 | 0 | 0 | 0 | 0 |  |
|  |  | 5 | 20 | 9,50 | 85,5 | 16 | 64 | 169,5 |
| **Option 2** | |  |  |  |  |  |  |  |
| 350 | ml full cream vanilla custard | 10 | 40 | 9 | 81 | 50 | 200 | 321 |
|  |  | 10 | 40 | 9 | 81 | 50 | 200 | 321 |
| **Option 3** | |  |  |  |  |  |  |  |
| 2 | knäckebröd golden brown | 3 | 12 | 1 | 9 | 12 | 48 | 69 |
| 1 | Topped with low-fat meat | 3 | 12 | 1 | 9 | 0 | 0 | 21 |
| 2 | with diet margarine | 0 | 0 | 7 | 63 | 0 | 0 | 63 |
| 1 | with jam | 0 | 0 | 0 | 0 | 9 | 36 | 36 |
| 1 | Glass of tea | 0 | 0 | 0 | 0 | 0 | 0 | 0 |
|  |  | 6 | 24 | 9 | 81 | 21 | 84 | 189 |
| **Option 4** | |  |  |  |  |  |  |  |
| 1 | Slice wheat bread | 3 | 12 | 1 | 9 | 16 | 64 | 85 |
| 1 | with diet margarine | 0 | 0 | 4 | 36 | 0 | 0 | 36 |
| 1 | with milk chocolate sprinkles | 1 | 4 | 2 | 18 | 11 | 44 | 66 |
| 1 | Cup of semi-skimmed milk | 7 | 28 | 3 | 27 | 10 | 40 | 95 |
|  |  | 11 | 44 | 10 | 90 | 37 | 148 | 282 |
| **Option 5** | |  |  |  |  |  |  |  |
| 300 | ml full- fat milk | 11 | 44 | 10 | 90 | 14 | 56 | 190 |
| 3 | Spoons of sugar | 0 | 0 | 0 | 0 | 15 | 60 | 60 |
| 30 | grams of bambix 8-wheat breakfast 15 months | 3 | 12 | 0 | 0 | 21 | 84 | 96 |
|  |  | 14 | 56 | 10 | 90 | 50 | 200 | 346 |
| **Option 6** | |  |  |  |  |  |  |  |
| 150 | ml full-fat yoghurt | 6 | 24 | 5 | 45 | 6 | 24 | 93 |
| 40 | gram muesli with sugar | 4 | 16 | 4 | 36 | 26 | 104 | 156 |
| 1 | Glass of tea | 0 | 0 | 0 | 0 | 0 | 0 | 0 |
|  |  | 10 | 40 | 9 | 81 | 32 | 128 | 249 |
| **Option 7** | |  |  |  |  |  |  |  |
| 1 | Slice wheat bread | 3 | 12 | 1 | 9 | 16 | 64 | 85 |
| 1 | with peanut butter | 4 | 16 | 8 | 72 | 2 | 8 | 96 |
| 1 | Glass of tea (with sugar) | 0 | 0 | 0 | 0 | 5 | 20 | 20 |
|  |  | 8 | 32 | 9 | 81 | 23 | 92 | 205 |
